# Gel‐Gel Interface Engineering for the Synthesis of Anisotropic Hydrogels with Designable Polymer Orientations

**DOI:** 10.1002/adma.202505268

**Published:** 2025-07-09

**Authors:** Tomohiro Takahashi, Naoya Karasawa, Koki Sano

**Affiliations:** ^1^ Department of Chemistry and Materials Faculty of Textile Science and Technology Shinshu University 3‐15‐1 Tokida Ueda Nagano 386–8567 Japan

**Keywords:** anisotropic hydrogels, gel adhesion, gel‐gel interfaces, photo‐patterning, polymer orientation

## Abstract

Anisotropic hydrogels with designable structural complexity can exhibit sophisticated properties reminiscent of those found in living organisms. However, conventional synthetic methods typically require specific anisotropic additives and complicated processes, limiting design flexibility. Here, a simple and versatile strategy to synthesize anisotropic hydrogels with photo‐designable orientations of polymer networks is developed by harnessing a “gel‐gel interface,” which can be generated through the intentional adhesion between hydrogels. This strategy originates from the serendipitous discovery: when a gel‐gel interface is intentionally introduced into a hydrogel through a two‐step polymerization, the polymer networks near the interface are spontaneously aligned perpendicular to the interface, as a result of the simultaneous swelling and fixation process during gel‐to‐gel adhesion. By employing a photo‐initiator system to control the gel‐gel interface, anisotropic hydrogels with both 2D and 3D designed polymer orientations, as well as anisotropic hydrogels with thermally switchable polymer orientations, are successfully synthesized. The gel‐gel interface has long been regarded as merely an undesirable byproduct of gel adhesion, while the complementary bulk region of hydrogels has been the primary focus in constructing anisotropic hydrogels. In contrast, this work demonstrates the utility of the gel‐gel interface, expanding design possibilities for next‐generation hydrogels with designable structural complexity.

## Graphical abstract

This study reports that when a gel‐gel interface is intentionally introduced into a hydrogel via two‐step polymerization, the polymer networks near the interface spontaneously align perpendicular to it. By employing a photo‐initiator system to spatially control the gel‐gel interface, anisotropic hydrogels with programmable orientations of polymer networks can be synthesized, thereby demonstrating the utility of the gel‐gel interface.

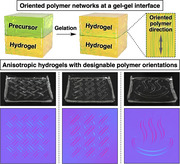

## 1 Introduction

Hydrogels are an important class of soft materials consisting of a 3D polymer network that retains a large amount of water. Due to their flexibility, biocompatibility, and compositional similarity to biological tissues, hydrogels have diverse applications in biomedical and biomimetic sciences, as well as in soft robotics and soft electronics.^[^ ^1^ ^]^ Recently, a fundamental difference in internal structures between artificial hydrogels and biological systems has been widely recognized.^[^ ^2^, ^3^, ^4^, ^5^, ^6^ ^]^ Conventional artificial hydrogels typically consist of isotropic polymer networks because polymerization or self‐assembly during hydrogelation usually occurs randomly. In contrast, most natural soft materials, such as muscle, skin, articular cartilage, and wood, are composed of anisotropically oriented structures, enabling their sophisticated functions. Inspired by these biological systems, various anisotropic hydrogels have been artificially synthesized primarily by aligning the constituent polymer networks or anisotropic additives throughout the bulk region of the hydrogel using external stimuli, such as mechanical forces,^[^ ^7^, ^8^, ^9^, ^10^, ^11^ ^]^ electric fields,^[^ ^12^, ^13^, ^14^ ^]^ magnetic fields,^[^ ^15^, ^16^, ^17^, ^18^, ^19^, ^20^, ^21^ ^]^ directional freezing,^[^ ^22^, ^23^, ^24^, ^25^, ^26^ ^]^ swelling,^[^ ^27^, ^28^, ^29^ ^]^ ion diffusion,^[^ ^30^, ^31^, ^32^, ^33^, ^34^, ^35^ ^]^ and shear flow.^[^ ^21^, ^36^, ^37^, ^38^, ^39^, ^40^, ^41^, ^42^, ^43^, ^44^ ^]^ Among them, anisotropic hydrogels with complex orientations, rather than simple unidirectional orientations, have gained increasing attention due to their unique properties, including programmable swelling,^[^ ^37^ ^]^ designable actuation,^[^ ^12^, ^13^, ^14^, ^15^, ^38^, ^42^ ^]^ self‐regulated motion,^[^ ^44^ ^]^ and mechanical nonreciprocity.^[^ ^20^ ^]^ However, it is typically necessary to use anisotropic additives for inducing the orientational complexity, such as nanosheets,^[^ ^12^, ^13^, ^14^, ^15^, ^20^, ^41^, ^44^ ^]^ nanofibers,^[^ ^37^, ^38^, ^42^ ^]^ and rigid polyelectrolytes,^[^ ^28^, ^34^ ^]^ restricting the range of available gel components. Moreover, the methods for achieving such complex orientations often require complicated multi‐step processes, which limit design flexibility. To overcome these challenges, there is a strong demand for a novel strategy that allows for the facile formation of designable structural orientations in hydrogels without relying on anisotropic additives.

During our studies, we serendipitously discovered a potential solution to these challenges by harnessing a “gel‐gel interface,” which is a complementary region to the bulk region of hydrogels. Generally, a gel‐gel interface can be generated through adhesion between two hydrogels, which results from the hybridization of gels,^[^ ^45^, ^46^, ^47^, ^48^, ^49^, ^50^, ^51^, ^52^, ^53^, ^54^, ^55^, ^56^, ^57^, ^58^, ^59^, ^60^, ^61^, ^62^, ^63^ ^]^ self‐healing of gels,^[^ ^64^, ^65^, ^66^, ^67^ ^]^ or 3D printing of gels.^[^ ^37^, ^38^, ^42^, ^68^, ^69^, ^70^, ^71^ ^]^ Most studies have focused on improving gel‐to‐gel adhesion by designing adhesive processes or molecular structures at the interfaces, whereas the meso‐ and macro‐scale structures of the polymer networks at the gel‐gel interface have been scarcely investigated. Additionally, there is little motivation to deliberately create a gel‐gel interface for purposes other than gel adhesion because it is often regarded as a defect that compromises various properties, including mechanical strength. Consequently, the gel‐gel interface has long been regarded as merely an undesirable byproduct of gel adhesion, and its potential utility has been overlooked, in contrast to the bulk region, which has been the primary focus in the synthesis of anisotropic hydrogels.

**Figure 1:**
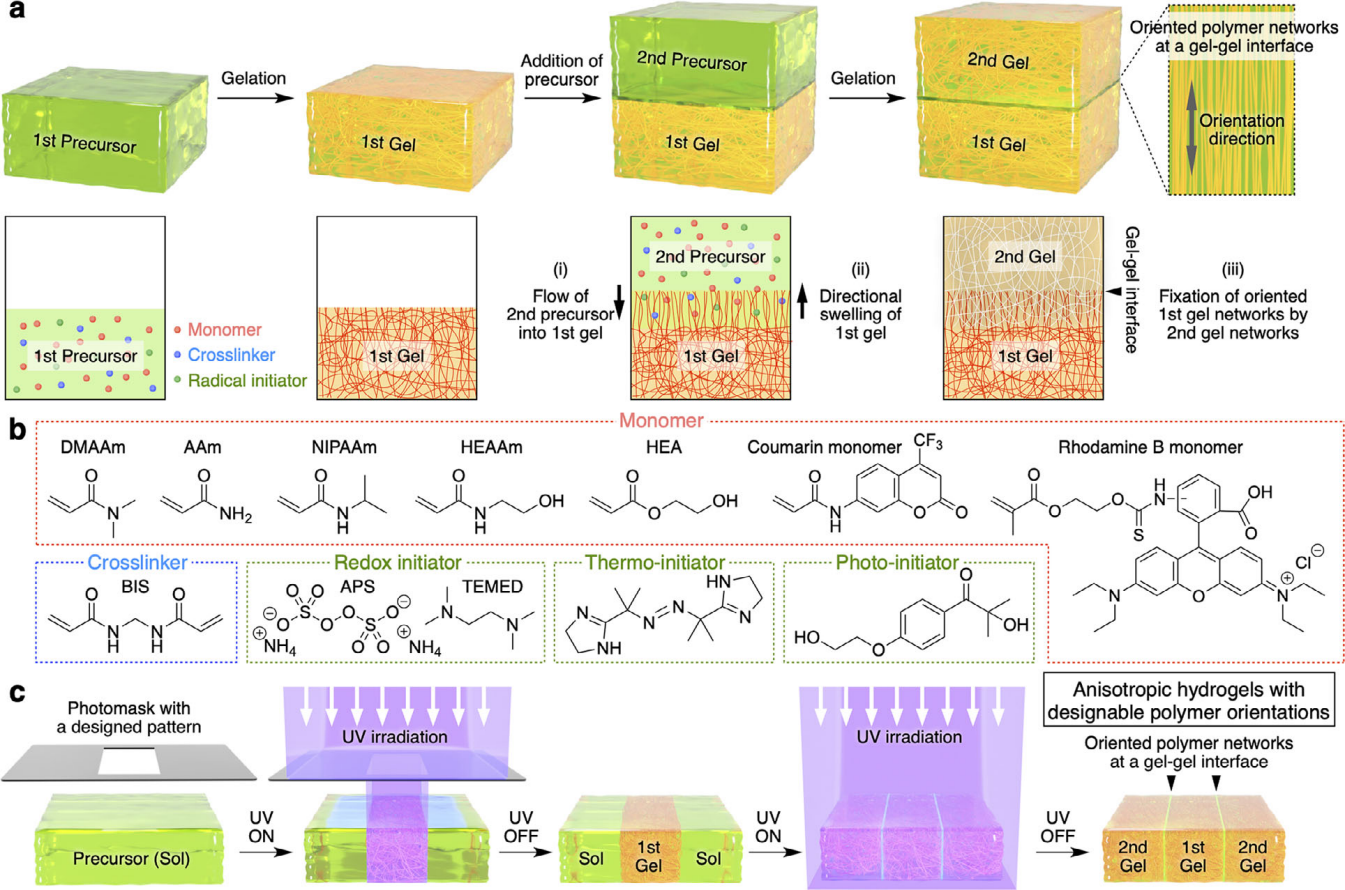
Spontaneous orientation of polymer networks through the introduction of a gel‐gel interface and a photo‐controlling method for anisotropic hydrogels with designable polymer orientations. a) Schematic illustrations of a method for introducing a gel‐gel interface within a hydrogel via a two‐step radical polymerization. b) Chemical structures of monomers, a crosslinker, and initiator systems. c) Schematic illustrations of a simple and versatile method for synthesizing anisotropic hydrogels with designable orientations of the polymer networks using a photo‐initiator system.

Contrary to this notion, in the present work, we intentionally introduced a gel‐gel interface into a hydrogel via a two‐step radical polymerization and discovered that the polymer networks near the gel‐gel interface were spontaneously aligned perpendicular to the interface (**Figure** **1**a). The mechanism behind this local orientation phenomenon can be explained by the simultaneous swelling and fixation process that occurs during the gel‐to‐gel adhesion. Due to the broad applicability of this mechanism, various types of monomers (acrylamide and acrylate monomers) and radical initiators (redox, thermo‐, and photo‐initiators) can be used (Figure 1b), unlike conventional synthetic methods for anisotropic hydrogels.^[^ ^2^, ^3^, ^4^, ^5^, ^6^, ^7^, ^8^, ^9^, ^10^, ^11^, ^12^, ^13^, ^14^, ^15^, ^16^, ^17^, ^18^, ^19^, ^20^, ^21^, ^22^, ^23^, ^24^, ^25^, ^26^, ^27^, ^28^, ^29^, ^30^, ^31^, ^32^, ^33^, ^34^, ^35^, ^36^, ^37^, ^38^, ^39^, ^40^, ^41^, ^42^, ^43^, ^44^ ^]^ Furthermore, by employing a photo‐initiator system to introduce well‐defined gel‐gel interfaces, we successfully established a universal method for synthesizing anisotropic hydrogels with designable orientations of polymer networks (Figure 1c). Notably, such polymer orientation controllability has not been achieved using conventional strategies for the synthesis of anisotropic hydrogels,^[^ ^2^, ^3^, ^4^, ^5^, ^6^, ^7^, ^8^, ^9^, ^10^, ^11^, ^12^, ^13^, ^14^, ^15^, ^16^, ^17^, ^18^, ^19^, ^20^, ^21^, ^22^, ^23^, ^24^, ^25^, ^26^, ^27^, ^28^, ^29^, ^30^, ^31^, ^32^, ^33^, ^34^, ^35^, ^36^, ^37^, ^38^, ^39^, ^40^, ^41^, ^42^, ^43^, ^44^ ^]^ nor by conventional photo‐patterning systems for hydrogels.^[^ ^28^, ^72^, ^73^, ^74^, ^75^, ^76^, ^77^ ^]^ By adjusting the gelation conditions, we successfully constructed anisotropic hydrogels with chiral arrangements of polymer networks and achieved reversible switching of the polymer orientation in a thermoresponsive anisotropic hydrogel by modulating temperature. This study highlights the utility of the gel‐gel interface in hydrogels, which has long been regarded as an undesirable byproduct of gel adhesion, and offers a complementary strategy to conventional bulk‐focused methods^[^ ^2^, ^3^, ^4^, ^5^, ^6^, ^7^, ^8^, ^9^, ^10^, ^11^, ^12^, ^13^, ^14^, ^15^, ^16^, ^17^, ^18^, ^19^, ^20^, ^21^, ^22^, ^23^, ^24^, ^25^, ^26^, ^27^, ^28^, ^29^, ^30^, ^31^, ^32^, ^33^, ^34^, ^35^, ^36^, ^37^, ^38^, ^39^, ^40^, ^41^, ^42^, ^43^, ^44^ ^]^ for synthesizing designable anisotropic hydrogels.

## 2 Results and Discussion

### 2.1 Preparation and Characterization of a Hydrogel with a Gel‐Gel Interface

**Figure 2:**
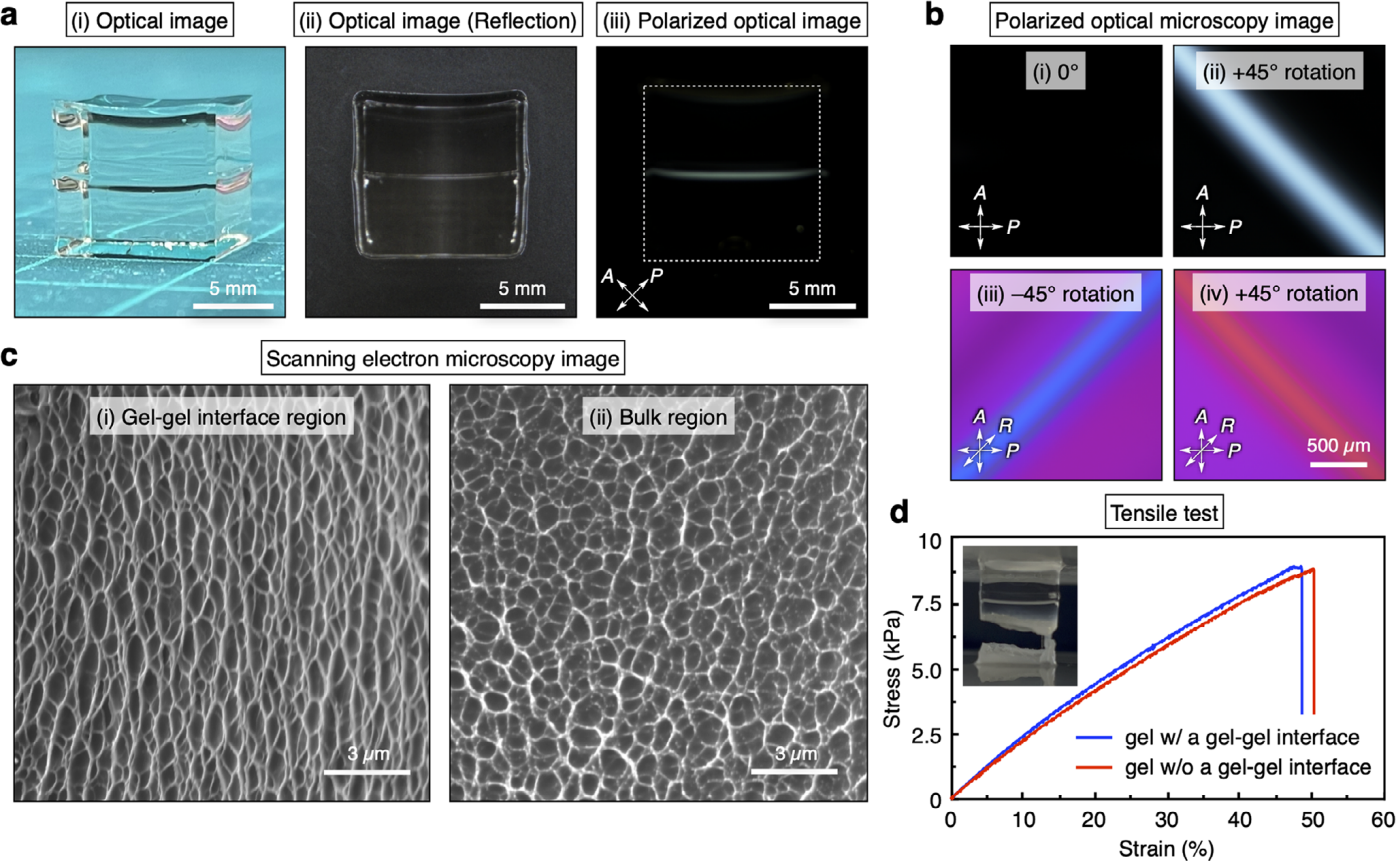
Structural characteristics of an anisotropic hydrogel with oriented polymer networks at a gel‐gel interface. a) (i) A photograph of overall structures of an anisotropic hydrogel with a gel‐gel interface (10 × 10 × 10 mm). (ii, iii) Optical (ii) and polarized optical images under crossed Nicols (iii) of the anisotropic hydrogel. b) Polarized optical microscopy images under crossed Nicols of a gel‐gel interface in the anisotropic hydrogel without (i and ii) and with (iii and iv) a sensitive tint plate. c) (i, ii) Field‐emission scanning electron microscopy images of the gel‐gel interface (i) and bulk (ii) regions in dried samples of the anisotropic hydrogel. d) Tensile stress‐strain curves of hydrogels with and without a gel‐gel interface. Inset: a photograph of an anisotropic hydrogel with a gel‐gel interface just after its fracture.

A gel‐gel interface in this work was typically introduced within a hydrogel through the following two‐step radical polymerization (Figure 1a). 1) An aqueous precursor solution containing a monomer (*N*,*N*‐dimethylacrylamide; DMAAm), a crosslinker (*N*,*N'*‐methylenebisacrylamide; BIS), and a redox initiator (ammonium persulfate; APS) was poured into a container (size: 10 × 10 × 45 mm) at room temperature. Then, an aqueous solution of *N*,*N*,*N'*,*N'*‐tetramethylethylenediamine (TEMED) was added to the precursor solution in the container to accelerate radical polymerization. After waiting for 5.0 min, the 1st gel (10 wt.% DMAAm, 0.10 wt.% BIS, 2.3 wt.% APS, and 0.2 wt.% TEMED) was formed. 2) The precursor solution of the same components (DMAAm, BIS, and APS) was poured on top of the 1st gel. After 15 s, the same TEMED solution was added to the precursor solution. The subsequent gelation for more than 5.0 min afforded a hydrogel with a gel‐gel interface, where the 1st and 2nd gels adhered firmly to each other. The gel‐gel interface within the resultant hydrogel could be visually detected (**Figure** **2**a). First, the orientation of the polymer networks near the gel‐gel interface was characterized by polarized optical microscopy (POM) under crossed Nicols, field‐emission scanning electron microscopy (FE‐SEM), and 2D small‐angle X‐ray scattering (2D‐SAXS). In the POM image of the hydrogel, bright birefringence was observed near the gel‐gel interface, indicating the local orientation of the polymer networks (Figure 2a‐iii). In contrast, other regions of the hydrogel remained dark, as typically observed in ordinary hydrogels with isotropic polymer networks. POM analysis with a sensitive tint plate (Figure 2b) suggested that the polymer networks were aligned perpendicular to the gel‐gel interface, based on comparison with a reference hydrogel (Figure S1, Supporting Information). FE‐SEM observations of the dried hydrogel further supported the perpendicular orientation of the polymer networks near the gel‐gel interface and the isotropic polymer networks in the bulk region (Figure 2c; Figure S2, Supporting Information). Additionally, as shown in the 2D‐SAXS data and the corresponding azimuthal angle plots (Figure S3, Supporting Information), an anisotropic scattering pattern was detected in the oriented polymer region, whereas an isotropic scattering pattern was observed in the bulk regions of both the 1st and 2nd gels. These 2D‐SAXS results are consistent with the POM and FE‐SEM observations. Interestingly, despite numerous studies on adhesion between hydrogels,^[^ ^45^, ^46^, ^47^, ^48^, ^49^, ^50^, ^51^, ^52^, ^53^, ^54^, ^55^, ^56^, ^57^, ^58^, ^59^, ^60^, ^61^, ^62^, ^63^ ^]^ such spontaneous polymer orientation at a gel‐gel interface has been overlooked, possibly because most studies have focused on achieving strong adhesion by designing adhesive processes or molecular structures, rather than thoroughly investigating the resultant meso‐ and macro‐scale structures of the polymer networks at the interface. It is noteworthy that the mechanical properties of the anisotropic hydrogel with the gel‐gel interface were almost comparable to those of a hydrogel without the interface, as shown in the stress‐strain curves (Figure 2d), indicating sufficient interfacial adhesion for use as an integrated hydrogel. Indeed, fracture during the tensile test occurred not at the gel‐gel interface but in the bulk region (Figure 2d, inset). As discussed in the next section, this sufficient adhesion between the 1st and 2nd gels can be attributed to topological crosslinking, where the polymer networks of the 1st and 2nd gels are physically entangled at the interface to form interpenetrating polymer networks.^[^ ^50^, ^51^, ^52^, ^53^ ^]^

### 2.2 Mechanism of Spontaneous Polymer Orientation near a Gel‐Gel Interface

**Figure 3:**
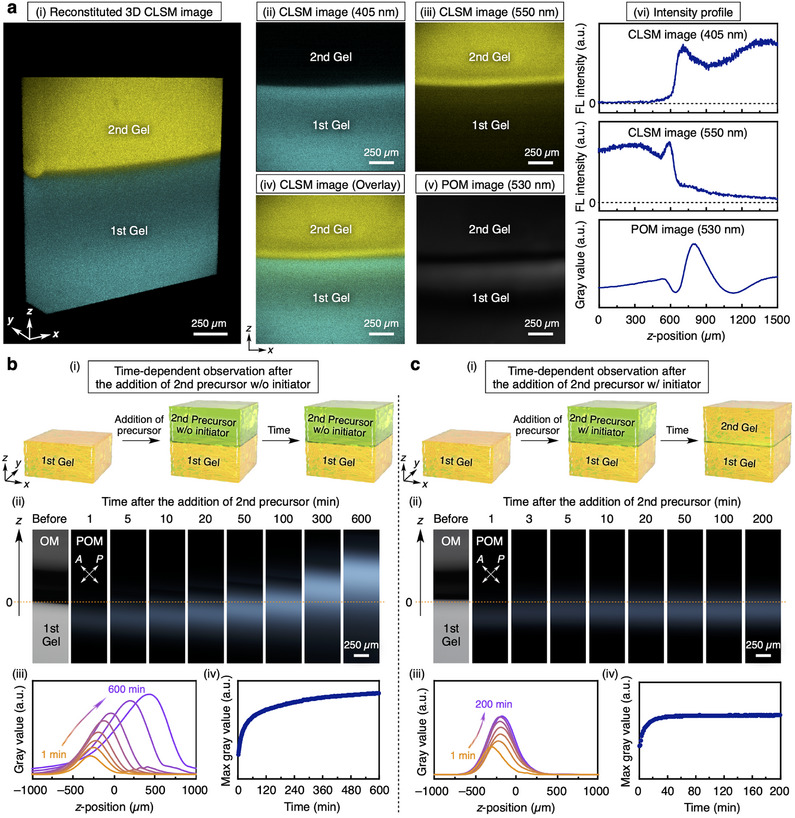
Formation mechanism of the spontaneous polymer orientation at a gel‐gel interface. a) (i) A reconstituted 3D confocal laser scanning microscopy (CLSM) image of an anisotropic hydrogel with a gel‐gel interface (405 nm laser for coumarin monomer in the 1st gel; 550 nm laser for rhodamine B monomer in the 2nd gel). (ii–iv) 2D CLSM images of the hydrogel visualized using a 405 nm laser (ii) and a 550 nm laser (iii), and their overlay CLSM image (iv). (v) A polarized optical microscopy (POM) image under crossed Nicols of the hydrogel visualized using a 530 nm laser. (vi) Intensity profiles along the z‐direction in the images (ii), (iii), and (v). b,c) (i–iv) Schematic illustrations (i), time‐dependent POM observations (ii), their intensity profiles along the z‐direction (iii), and time‐dependent profiles of their maximum gray values (iv) of the gel‐gel interface after the addition of a precursor solution for the 2nd gel without (b) and with (c) the radical initiator.

To explain the formation mechanism of the local orientation, we further characterized the internal structures of the hydrogel with a gel‐gel interface by performing confocal laser scanning microscopy (CLSM) observations in addition to time‐dependent POM observations. First, we visualized the polymer networks of the 1st and 2nd gels by using two different fluorescent monomers (coumarin and rhodamine B monomers in Figure 1b). The CLSM and POM observations of the hydrogel in **Figure** **3**a led to the following two findings: 1) In the CLSM images, the fluorescence intensity of the 1st gel suddenly dropped at the gel‐gel interface and it was not observed in the 2nd gel region (Figure 3a‐ii), whereas that of the 2nd gel decreased gradually across the gel‐gel interface, with fluorescence detected even in the 1st gel region (Figure 3a‐iii). 2) The bright birefringence of the oriented polymer networks in the POM image was observed in the 1st gel but not in the 2nd gel (Figure 3a‐v), consistent with the FE‐SEM observations (Figure S2, Supporting Information). These two findings suggest that the spontaneous polymer orientation may occur through the following swelling and fixation process. **Swelling process**: As a result of the inflow of the precursor solution for the 2nd gel into the 1st gel during radical polymerization (Figure 1a‐i), the interface region of the 1st gel swelled directionally, locally aligning the polymer networks of the 1st gel at the interface (Figure 1a‐ii). **Fixation process**: Simultaneously, the gelation of the precursor solution for the 2nd gel took place in both the 1st and 2nd gel regions, fixing this local orientation of the polymer networks (Figure 1a‐iii). Due to this simultaneous swelling and fixation process, the polymer networks of the 1st and 2nd gels become physically entangled near the gel‐gel interface to form interpenetrating polymer networks, resulting in topological crosslinking that ensures their strong adhesion.^[^ ^50^, ^51^, ^52^, ^53^ ^]^

To further support the proposed mechanism (i.e., swelling and fixation process), we conducted time‐dependent POM observations during the formation of the gel‐gel interface, both in the presence and absence of the radical initiator (APS/TEMED). In the absence of the initiator, the POM observations revealed that the front of the oriented polymer region continuously propagated along the *z*‐direction for more than 600 min, and the birefringence increased over time (Figure 3b; Movie S1, Supplementary Movie1). This behavior indicates directional swelling of the 1st gel and the resultant orientation of its polymer networks. In contrast, in the presence of the initiator, the birefringence at the gel‐gel interface initially increased and became constant after ≈60 min, accompanied by the suppression of further directional swelling of the 1st gel (Figure 3c; Movie S2, Supplementary Movie2). These observations suggest that the oriented polymer networks of the 1st gel were fixed through gelation of the precursor solution for the 2nd gel in both the 1st and 2nd gel regions to form the interpenetrating polymer networks. These findings further support the proposed formation mechanism of the spontaneously oriented polymer networks via the simultaneous swelling and fixation process at the gel‐gel interface. Importantly, the fixation process plays a crucial role not only in stabilizing the oriented polymer networks that continuously change during the directional swelling, but also in embedding the oriented polymer networks, originally confined to the gel surface, into the interior of the gel.

### 2.3 Controllability and Generality of the Spontaneous Polymer Orientation at a Gel‐Gel Interface

**Figure 4:**
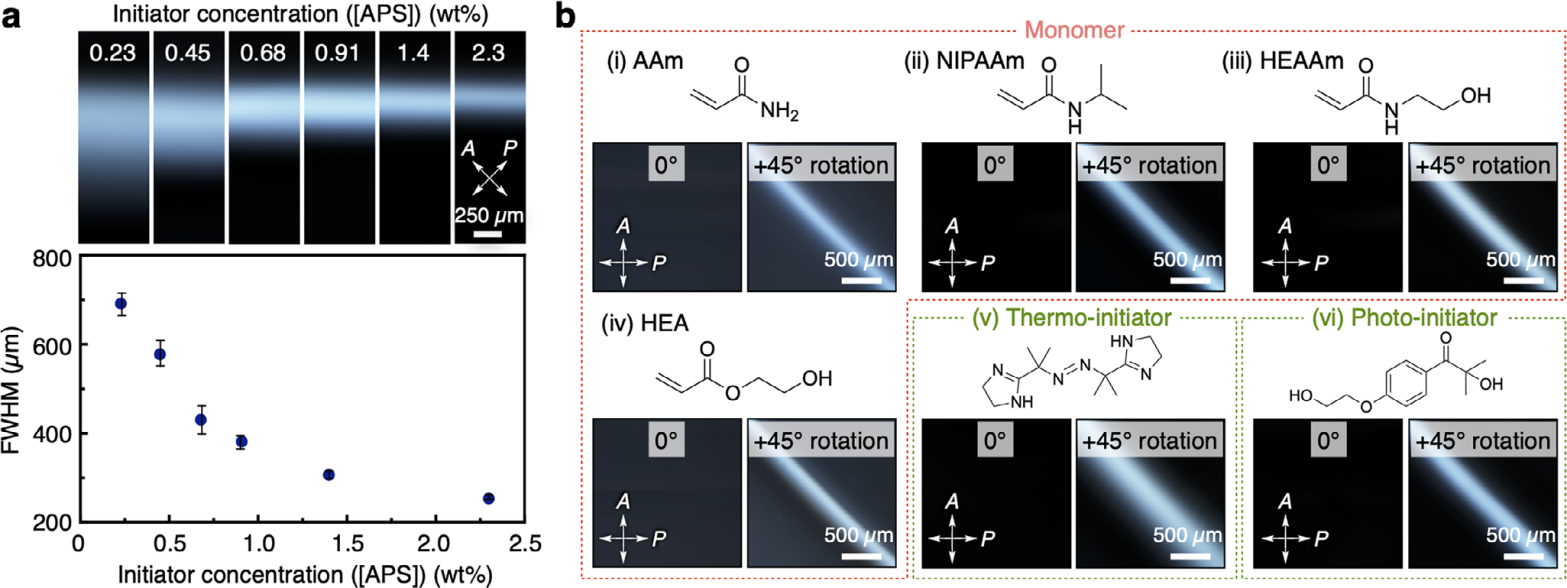
Controllability and generality of the spontaneous polymer orientation at a gel‐gel interface. a) Polarized optical microscopy images under crossed Nicols (upper) of the gel‐gel interface in anisotropic hydrogels using different initiator concentrations ([APS] = 0.23, 0.45, 0.68, 0.91, 1.4, and 2.3 wt.%) and the plots of the full width at half maximum (FWHM) in their birefringence profiles (lower). Data are presented as mean values ± SD (n = 6). b) Polarized optical microscopy images under crossed Nicols of the gel‐gel interface in anisotropic hydrogels with different types of monomers [(i) acrylamide (AAm), (ii) *N*‐isopropylacrylamide (NIPAAm), (iii) *N*‐(2‐hydroxyethyl)acrylamide (HEAAm), (iv) 2‐hydroxyethyl acrylate (HEA)] and initiators [(v) thermo‐ and (vi) photo‐initiators].

Considering the role of swelling in the polymer orientation mechanism, we hypothesized that the size of the oriented polymer region could be controlled by adjusting the swelling time. Since the swelling time is governed by the kinetics of the fixation process (i.e., the gelation time), the concentration of the radical initiator ([APS]) was expected to be a key parameter. Accordingly, we performed time‐resolved FT‐IR spectroscopy^[^ ^78^ ^]^ and rheological measurements to investigate the effects of [APS] on the polymerization rate and gelation time, respectively. The results revealed that decreasing [APS] led to both a slower polymerization rate and a longer gelation time (Figures S4, S5, Supporting Information). Subsequently, we confirmed that the size of the locally oriented polymer region, evaluated by the full width at half maximum (FWHM) of the birefringence profile, increased from 254 to 692 µm by varying [APS] from 2.3 to 0.23 wt.% (**Figure** **4**a). Furthermore, given the universality of both the swelling and fixation processes, we anticipated that the spontaneous polymer orientation would be independent of the gel components. Indeed, even when various monomers (acrylamide, *N*‐isopropylacrylamide, *N*‐(2‐hydroxyethyl)acrylamide, 2‐hydroxyethyl acrylate) and initiators (thermo and photo‐initiators) were used (Figure 1b), the birefringence derived from oriented polymer networks was also observed near the gel‐gel interface in the POM images (Figure 4b). These results demonstrate the universality of this system, offering a simple and versatile strategy for synthesizing designable anisotropic hydrogels without the need for anisotropic additives, in contrast to conventional approaches.

### 2.4 Synthesis of Anisotropic Hydrogels with Designed Orientations of Polymer Networks

**Figure 5:**
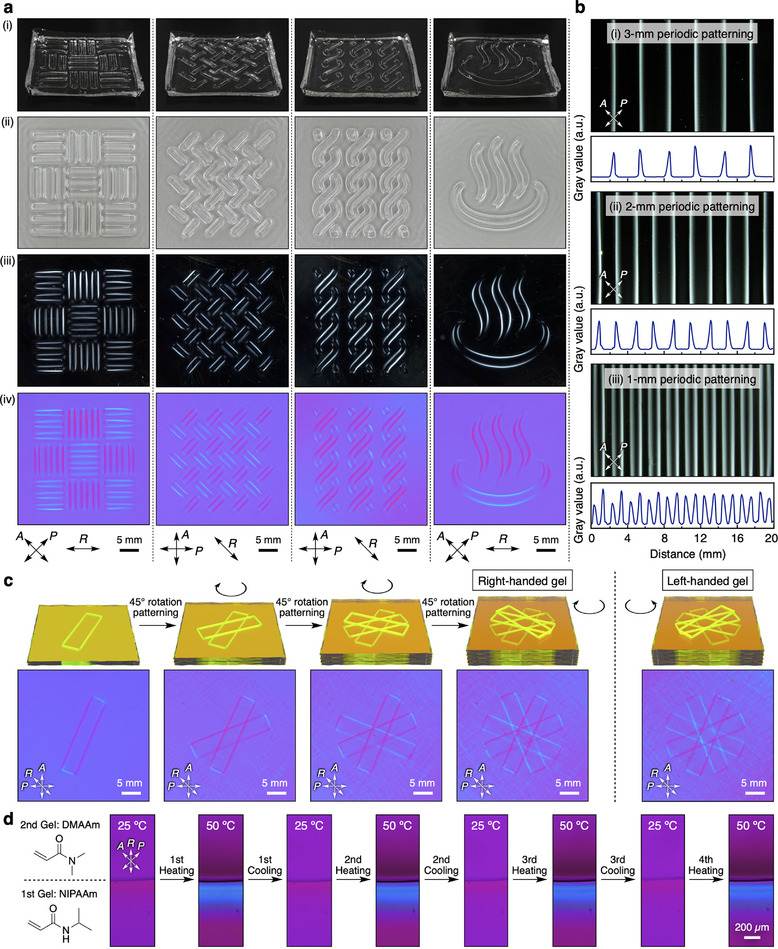
Synthesis of anisotropic hydrogels with designed orientations of polymer networks using a photo‐induced gel‐gel interface. a) (i) Photographs of the overall structures of anisotropic hydrogels with designable orientations of polymer networks (30 × 30 × 1.7 mm). (ii–iv) Optical (ii) and polarized optical top‐view images under crossed Nicols without (iii) and with (iv) a sensitive tint plate of the anisotropic hydrogels. b) (i–iii) Polarized optical images (upper) and the corresponding gray value profiles (lower) of anisotropic hydrogels with periodic patterning of 3 mm (i), 2 mm (ii), and 1 mm (iii). c) Polarized optical images with a sensitive tint plate of anisotropic hydrogels with chiral arrangements of polymer networks synthesized via a step‐by‐step photo‐patterning process. d) Reversible switching of the polymer network orientation in an anisotropic hydrogel consisting of thermoresponsive polymer networks (1st gel: NIPAAm; 2nd gel: DMAAm) in response to temperature changes between 25 and 50 °C.

As the polymer orientation at the gel‐gel interface can be successfully induced using a photo‐initiator, we came up with the idea of using light to control the orientation of polymer networks by introducing the gel‐gel interface with a designed shape (Figure 1c). First, an aqueous precursor solution containing the photo‐initiator was poured into a container, and the solution was irradiated with UV light through a photomask with a designed pattern (Figure S6, Supporting Information) for 2.5 min to induce gelation. Then, only the light‐irradiated region was converted into a hydrogel (1st gel), where the 1st gel interface was in contact with the precursor solution in the non‐irradiated region. Consequently, the interface of the 1st gel swelled directionally due to the inflow of the precursor solution in the non‐irradiated region, aligning its polymer networks (swelling process). The subsequent UV light irradiation, after the photomask was removed, induced gelation in other regions to form the 2nd gel, fixing the local orientation of the polymer networks of the 1st gel (fixation process). As a result, the oriented polymer networks, with the same shape as the boundary between the light‐transmitting and light‐blocking regions of the photomask, were embedded within the hydrogel. By performing these processes using photomasks with designed patterns (Figure S6, Supporting Information), we succeeded in synthesizing anisotropic hydrogels with designed orientations of polymer networks (**Figure** **5**a). Their POM images (Figure 5a‐iii), in conjunction with those obtained using a sensitive tint plate (Figure 5a‐iv), suggest that polymer networks were aligned perpendicular to the designed gel‐gel interfaces (i.e., birefringent lines in the POM images; Figure S7, Supporting Information), similar to the oriented polymer networks in the hydrogel with a gel‐gel interface (Figure 2). Importantly, this synthetic method enables the facile formation of both straight and curved sharp lines of the oriented polymer networks within hydrogels. Furthermore, anisotropic hydrogels with periodic patterns of 1‐, 2‐, and 3‐mm spacing were readily synthesized, demonstrating the high spatial resolution of this method (Figure 5b). Such designability of the oriented polymer networks in the hydrogels could not be achieved using conventional strategies for the synthesis of anisotropic hydrogels,^[^ ^2^, ^3^, ^4^, ^5^, ^6^, ^7^, ^8^, ^9^, ^10^, ^11^, ^12^, ^13^, ^14^, ^15^, ^16^, ^17^, ^18^, ^19^, ^20^, ^21^, ^22^, ^23^, ^24^, ^25^, ^26^, ^27^, ^28^, ^29^, ^30^, ^31^, ^32^, ^33^, ^34^, ^35^, ^36^, ^37^, ^38^, ^39^, ^40^, ^41^, ^42^, ^43^, ^44^ ^]^ nor by conventional photo‐patterning systems for hydrogels.^[^ ^28^, ^72^, ^73^, ^74^, ^75^, ^76^, ^77^ ^]^ Upon decreasing the photo‐initiator concentration or the UV irradiation time, the birefringence at the gel‐gel interface decreased, possibly due to a reduced degree of polymer orientation and/or incomplete gelation (Figure S8, Supporting Information).

Next, we attempted to synthesize anisotropic hydrogels with designed polymer orientations in 3D by repeating the synthetic process along the *z*‐direction. First, we performed the same gelation process using a photomask with a rectangular pattern, resulting in an anisotropic hydrogel with a rectangular orientation of polymer networks (Figure 5c). Then, a similar gelation process was applied to the resultant hydrogel using the photomask rotated 45° clockwise, affording an anisotropic hydrogel with a 3D orientation of polymer networks. By repeating these processes, we were able to synthesize an anisotropic hydrogel with a chiral arrangement of polymer networks (right‐handed gel in Figure 5c). The opposite‐handed chiral arrangement was also realized by repeating the same processes in a counterclockwise manner (left‐handed gel in Figure 5c and Figure S9, Supporting Information). Finally, we employed *N*‐isopropylacrylamide monomer to synthesize an anisotropic hydrogel composed of poly(*N*‐isopropylacrylamide), which is widely known for its thermoresponsive behavior at the lower critical solution temperature (LCST).^[^ ^79^ ^]^ Due to thermoresponsive shrinking and swelling of the local polymer networks driven by changes in their hydrophilic‐hydrophobic balance, we successfully achieved reversible switching of the polymer orientation at the gel‐gel interface in the thermoresponsive hydrogel by modulating temperature between 25 and 50 °C (Figure 5d). Such reversible switching could lead to the control of diverse properties, including mechanical properties, actuation behaviors, and even thermal properties.^[^ ^80^, ^81^ ^]^

## 3 Conclusion

In summary, we have developed a simple and versatile strategy to synthesize anisotropic hydrogels with photo‐designable orientations of polymer networks by harnessing a “gel‐gel interface,” which can be generated through the intentional adhesion between hydrogels. Unlike conventional methods, this strategy does not require anisotropic additives and can be applied to a wide range of monomers and radical initiator systems. Furthermore, this strategy, using a photo‐initiator system, enables well‐defined photo‐control of polymer orientations in hydrogels, which could not be achieved using conventional strategies for the synthesis of anisotropic hydrogels,^[^ ^2^, ^3^, ^4^, ^5^, ^6^, ^7^, ^8^, ^9^, ^10^, ^11^, ^12^, ^13^, ^14^, ^15^, ^16^, ^17^, ^18^, ^19^, ^20^, ^21^, ^22^, ^23^, ^24^, ^25^, ^26^, ^27^, ^28^, ^29^, ^30^, ^31^, ^32^, ^33^, ^34^, ^35^, ^36^, ^37^, ^38^, ^39^, ^40^, ^41^, ^42^, ^43^, ^44^ ^]^ nor through conventional photo‐patterning systems for hydrogels.^[^ ^28^, ^72^, ^73^, ^74^, ^75^, ^76^, ^77^ ^]^ The development of this strategy originated from our unexpected discovery: when a gel‐gel interface was introduced into a hydrogel through a two‐step radical polymerization, the polymer networks near the interface were spontaneously aligned perpendicular to the interface, driven by the simultaneous swelling and fixation process during gel‐to‐gel adhesion. Interestingly, despite numerous studies on adhesion between hydrogels,^[^ ^45^, ^46^, ^47^, ^48^, ^49^, ^50^, ^51^, ^52^, ^53^, ^54^, ^55^, ^56^, ^57^, ^58^, ^59^, ^60^, ^61^, ^62^, ^63^ ^]^ such spontaneous polymer orientation at a gel‐gel interface has been overlooked. The gel‐gel interface has long been considered merely an undesirable byproduct of gel adhesion and its utility has been underestimated, while the bulk region of hydrogels has been the primary focus in synthesizing anisotropic hydrogels.^[^ ^2^, ^3^, ^4^, ^5^, ^6^, ^7^, ^8^, ^9^, ^10^, ^11^, ^12^, ^13^, ^14^, ^15^, ^16^, ^17^, ^18^, ^19^, ^20^, ^21^, ^22^, ^23^, ^24^, ^25^, ^26^, ^27^, ^28^, ^29^, ^30^, ^31^, ^32^, ^33^, ^34^, ^35^, ^36^, ^37^, ^38^, ^39^, ^40^, ^41^, ^42^, ^43^, ^44^ ^]^ In contrast to this notion, this work highlights the valuable role of the gel‐gel interface and provides a complementary strategy to the conventional bulk‐focused methods.^[^ ^2^, ^3^, ^4^, ^5^, ^6^, ^7^, ^8^, ^9^, ^10^, ^11^, ^12^, ^13^, ^14^, ^15^, ^16^, ^17^, ^18^, ^19^, ^20^, ^21^, ^22^, ^23^, ^24^, ^25^, ^26^, ^27^, ^28^, ^29^, ^30^, ^31^, ^32^, ^33^, ^34^, ^35^, ^36^, ^37^, ^38^, ^39^, ^40^, ^41^, ^42^, ^43^, ^44^ ^]^ The formation of a gel‐gel interface is a universal phenomenon in hydrogel engineering, including the hybridization of gels,^[^ ^45^, ^46^, ^47^, ^48^, ^49^, ^50^, ^51^, ^52^, ^53^, ^54^, ^55^, ^56^, ^57^, ^58^, ^59^, ^60^, ^61^, ^62^, ^63^ ^]^ self‐healing of gels,^[^ ^64^, ^65^, ^66^, ^67^ ^]^ and 3D printing of gels.^[^ ^37^, ^38^, ^42^, ^68^, ^69^, ^70^, ^71^ ^]^ Furthermore, considering its wide applicability, the strategy can be extended to other soft material systems and integrated with advanced engineering techniques, as well as conventional synthetic methods,^[^ ^2^, ^3^, ^4^, ^5^, ^6^, ^7^, ^8^, ^9^, ^10^, ^11^, ^12^, ^13^, ^14^, ^15^, ^16^, ^17^, ^18^, ^19^, ^20^, ^21^, ^22^, ^23^, ^24^, ^25^, ^26^, ^27^, ^28^, ^29^, ^30^, ^31^, ^32^, ^33^, ^34^, ^35^, ^36^, ^37^, ^38^, ^39^, ^40^, ^41^, ^42^, ^43^, ^44^ ^]^ for anisotropic hydrogels. Therefore, we believe that the present work will not only expand the design possibilities but also enhance the functionality of anisotropic soft materials, paving the way for the development of next‐generation smart materials with designable multi‐scale structural complexity.

## 4 Experimental Section

### Materials

*N*,*N*‐Dimethylacrylamide (DMAAm), *N*,*N'*‐methylenebisacrylamide (BIS), ammonium persulfate (APS), *N*,*N*,*N'*,*N'*‐tetramethylethylenediamine (TEMED), acrylamide (AAm), *N*‐(2‐hydroxyethyl)acrylamide (HEAAm), 2‐hydroxyethyl acrylate (HEA), and 2‐hydroxy‐4′‐(2‐hydroxyethoxy)‐2‐methylpropiophenone (photo‐initiator) were purchased from Tokyo Chemical Industry (TCI). *N*‐Isopropylacrylamide (NIPAAm), 2,2′‐azobis[2‐(2‐imidazolin‐2‐yl)propane] dihydrochloride (thermo‐initiator), ethanol, and tert‐butanol were purchased from FUJIFILM Wako Pure Chemical Corporation. 7‐[4‐(Trifluoromethyl)coumarin]acrylamide (coumarin monomer) and methacryloxyethyl thiocarbamoyl rhodamine B (rhodamine B monomer) were purchased from Sigma‐Aldrich and Polysciences, respectively. Ultrapure water, obtained from a Millipore model Milli‐Q IQ 7003 water purification system, was used throughout the experiments. Polymerization inhibitors in monomers were removed by using inhibitor removers (Sigma‐Aldrich) before use. Unless otherwise noted, all reagents were used as received from the commercial suppliers, after inhibitor removal if applicable.

### Preparation Method of a Hydrogel with a Gel‐Gel Interface

An anisotropic hydrogel with a gel‐gel interface was typically synthesized by the following procedure (Figure 2a,b,d). An aqueous precursor solution ([DMAAm] = 13 wt.%, [BIS] = 0.13 wt.%, [APS] = 3.0 wt.%, 375 µL) was poured into a polystyrene container (size: 10 × 10 × 45 mm) at room temperature. Then, an aqueous TEMED solution ([TEMED] = 0.8 wt.%, 125 µL) was added to the precursor solution in the container and quickly mixed to accelerate radical polymerization. After waiting for 5.0 min, the solution was converted to a hydrogel in the container. Next, the precursor solution of the same components ([DMAAm] = 13 wt.%, [BIS] = 0.13 wt.%, [APS] = 3.0 wt.%, 375 µL) was poured on top of the resultant hydrogel. After 15 s, the TEMED solution ([TEMED] = 0.8 wt.%, 125 µL) was added to the precursor solution and quickly mixed. The subsequent gelation for more than 5.0 min afforded a hydrogel with a gel‐gel interface ([DMAAm] = 10 wt.%, [BIS] = 0.10 wt.%, [APS] = 2.3 wt.%, [TEMED] = 0.2 wt.%). An anisotropic hydrogel with a gel‐gel interface composed of other monomers (AAm for Figure 4b‐i; HEAAm for Figure 4b‐iii; HEA for Figure 4b‐iv) was similarly synthesized by the replacement of DMAAm with the monomer. In the case of NIPAAm (Figure 4b‐ii), the mixing and radical polymerization processes were similarly performed in the ice bath and at room temperature, respectively. The size of the locally oriented polymer region (Figure 4a) was controlled by tuning the gelation time based on the initiator concentration ([APS]), as described below. The 1st gel ([DMAAm] = 10 wt.%, [BIS] = 0.10 wt.%, [APS] = 0.23, 0.45, 0.68, 0.91, 1.4, and 2.3 wt.%, [TEMED] = 0.2 wt.%) in a polystyrene container (size: 10 × 10 × 45 mm) was similarly synthesized, and after waiting for 50, 30, 20, 15, 10, and 5.0 min, respectively, to complete hydrogelation, the 375 µL precursor solution without TEMED was poured on top of the resultant hydrogel. After 15 s, the TEMED solution ([TEMED] = 0.8 wt.%, 125 µL) was added to the precursor solution and quickly mixed. The subsequent gelation overnight afforded a hydrogel containing a different size of locally oriented polymer region at a gel‐gel interface ([DMAAm] = 10 wt.%, [BIS] = 0.10 wt.%, [APS] = 0.23, 0.45, 0.68, 0.91, 1.4, and 2.3 wt.%, [TEMED] = 0.2 wt.%).

In the case of the thermo‐initiator system (Figure 4b‐v), an aqueous precursor solution ([DMAAm] = 10 wt.%, [BIS] = 0.10 wt.%, [thermo‐initiator] = 0.75 wt.%, 500 µL) was poured into a polystyrene container (size: 10 × 10 × 45 mm) and placed on a hot stage at 80 °C for 5.0 min to complete hydrogelation. Then, the container was cooled to room temperature, and the same precursor solution was poured on top of the resultant hydrogel. Subsequently, the container was heated again at 80 °C for 5.0 min, and finally, the container was cooled to room temperature. In the case of the photo‐initiator system (Figure 4b‐vi), an aqueous precursor solution ([DMAAm] = 10 wt.%, [BIS] = 0.10 wt.%, [photo‐initiator] = 0.25 wt.%, 500 µL) was poured into a polystyrene container (size: 10 × 10 × 45 mm), and the sample was irradiated with UV light for 5.0 min by using an USHIO model OPM2‐502H super high‐pressure mercury lamp (500 W) to complete hydrogelation. Then, the same precursor solution was poured on top of the resultant hydrogel, and after 1.0 min, the sample was irradiated again with UV light for 5.0 min.

A thermoresponsive hydrogel with switchable polymer orientations was synthesized by the following procedure (Figure 5d). First, an aqueous precursor solution ([NIPAAm] = 9.0 wt.%, [AAm] = 1.0 wt.%, [BIS] = 0.30 wt.%, [photo‐initiator] = 0.25 wt.%, 50 µL) was poured into a 0.5 mm‐thick quartz container, and the sample was irradiated with UV light for 5.0 min by using a Thorlabs model SOLIS‐365C high‐power LED light (365 nm, 4.0 W). Subsequently, another aqueous precursor solution ([DMAAm] = 10 wt.%, [BIS] = 0.10 wt.%, [photo‐initiator] = 0.25 wt.%, 50 µL) was poured into the container. After waiting for 10 min, the sample was irradiated again with UV light for 5.0 min.

### Optical and Polarized Optical Observations

Optical and polarized optical images (Figure 2a‐ii,‐iii; Figure 5b; Figure S8, Supporting Information) and polarized optical microscopy images (Figure 2b; Figure 3b‐ii,c‐ii; Figure 4; Figure S1, Supporting Information; Movies S1 and S2, Supplementary Movie1 and Movie2) of the hydrogels with gel‐gel interfaces were taken at room temperature by using a Nikon model SMZ 1270i Zoom Stereo Microscope and a Nikon model Eclipse LV100N POL optical polarizing microscope, respectively. In Figure 5d, polarized optical microscopy images were taken at 25 and 50 °C with a heating/cooling rate of 25 °C min^−1^ by using a Nikon model Eclipse LV100N POL optical polarizing microscope equipped with a Linkam model 10030 temperature controller. Polarized optical images (Figure 5a‐iii,‐iv; Figure 5c; Figure S9, Supporting Information) of the anisotropic hydrogels with designed polymer orientations were observed at room temperature on a MeCan Imaging model MRV‐ST Polariscope.

### Field‐Emission Scanning Electron Microscopy (FE‐SEM) Observations

FE‐SEM observations of a freeze‐dried hydrogel with a gel‐gel interface were performed on a JEOL model JSM‐IT800SHL. The freeze‐dried hydrogel was prepared using the following method. The hydrogel with a gel‐gel interface ([DMAAm] = 10 wt.%, [BIS] = 1.0 wt.%, [TEMED] = 0.2 wt.%, [APS] = 2.3 wt.% for Figure 2c and [APS] = 0.23 wt.% for Figure S2, Supporting Information) was sliced and immersed in ultrapure water for washing. Next, the internal solvent of the washed hydrogel was gradually replaced with 100% ethanol, and then gradually replaced again with 100% tert‐butanol. Finally, the resultant gel was rapidly frozen at −30 °C and freeze‐dried for more than 30 h using an EYELA model FD‐1000 freeze dryer. The region near the gel‐gel interface and the bulk region of the freeze‐dried gel were cut, and the samples were then observed after osmium coating.

### 2D Small‐Angle X‐ray Scattering (2D‐SAXS) Measurements

2D‐SAXS measurements were performed using a Rigaku model NANOPIX 3.5m system equipped with a Rigaku model HyPix‐6000 detector (X‐ray wavelength: 1.54 Å; sample‐to‐detector distance: 894 mm). The freeze‐dried gel sample ([DMAAm] = 10 wt.%, [BIS] = 1.0 wt.%, [TEMED] = 0.2 wt.%, [APS] = 0.23 wt.%) was prepared under the same conditions as those used for FE‐SEM observations. Azimuthal angle plots were obtained from the corresponding scattering patterns using the Rigaku 2DP software (Figure S3, Supporting Information).

### Tensile Tests

Tensile tests were carried out at room temperature by using a Shimadzu model EZ test tensile tester with a 50 N load cell (Figure 2d). The top and bottom faces of the hydrogel with a gel‐gel interface ([DMAAm] = 10 wt.%, [BIS] = 0.10 wt.%, [APS] = 2.3 wt.%, [TEMED] = 0.2 wt.%, size: 10 mm cubic) were glued to the upper and lower plates of the tensile tester, and tensile stress was measured at a constant tensile rate of 3 mm min^−1^. A hydrogel without a gel‐gel interface was similarly synthesized, except for the two‐step gelation process, and its tensile test was also conducted in the same manner.

### Confocal Laser Scanning Microscopy (CLSM) Observations

Confocal laser scanning microscopy (CLSM) observations of the anisotropic hydrogel with a gel‐gel interface were performed at 25 °C on a Leica STELLARIS 8 confocal microscope platform (**Figure** **3**a). The hydrogel was prepared using the following method. An aqueous precursor solution ([DMAAm] = 10 wt.%, [coumarin monomer] = 0.0013 wt.%, [BIS] = 0.10 wt.%, [APS] = 2.3 wt.%, [TEMED] = 0.2 wt.%, 80 µL) was rapidly poured into a 1 mm‐thick quartz container at room temperature. After waiting for 5.0 min for complete hydrogelation, the container surface was washed repeatedly to remove the dye, and a precursor solution containing a different dye monomer ([DMAAm] = 10 wt.%, [rhodamine B monomer] = 0.0060 wt.%, [BIS] = 0.10 wt.%, [APS] = 2.3 wt.%, [TEMED] = 0.2 wt.%, 80 µL) was poured on top of the 1st gel. After the gelation for more than 30 min, the resultant hydrogel film with a thickness of 1 mm was removed from the container, and the film was sandwiched between cover glasses for CLSM observations. The 1st and 2nd gels were visualized by CLSM using 405 and 550 nm lasers, and these 2D images were collected with a *z*‐step size of 2 µm. The obtained 2D images were reconstituted to a thickness of 300 µm to provide 3D information of the hydrogel. A polarized optical microscopy (POM) image under crossed Nicols of the hydrogel was obtained in a transmittance mode of CLSM using a 530 nm laser. Intensity profiles (Figure 3a‐vi) along the *z*‐direction in the CLSM and POM images (Figure 3a‐ii,‐iii,‐v) were obtained by using ImageJ (Fiji).

### Time‐Dependent Polarized Optical Microscopy (POM) Observations

Time‐dependent polarized optical microscopy (POM) images under crossed Nicols were taken every minute at room temperature by using a Nikon model Eclipse LV100N POL optical polarizing microscope as follows (Figure 3b,c; Movies S1 and S2, Supplementary Movie1 and Movie2). After the formation of the 1st gel ([DMAAm] = 10 wt.%, [BIS] = 0.10 wt.%, [APS] = 2.3 wt.%, [TEMED] = 0.2 wt.%) in a 10 mm‐thick quartz cuvette, a precursor solution with a radical initiator ([DMAAm] = 10 wt.%, [BIS] = 0.10 wt.%, [APS] = 0.68 wt.%, [TEMED] = 0.2 wt.%; Figure 3c) or without the initiator ([DMAAm] = 10 wt.%, [BIS] = 0.10 wt.%; Figure 3b) was poured into the cuvette so that the hydrogel interface was fully immersed in the precursor solution. Then, the time‐dependent changes in the region near the interface were monitored by POM images. Intensity profiles (Figure 3b‐iii,c‐iii) along the *z*‐direction in the POM images (Figure 3b‐ii,c‐ii) were obtained by using ImageJ (Fiji).

### Preparation Method of Anisotropic Hydrogels with Designable Polymer Orientations

Anisotropic hydrogels with designable polymer orientations were typically synthesized by the following procedure (Figure 5a‐ii,‐iii,‐iv). First, an aqueous precursor solution ([DMAAm] = 17 wt.%, [BIS] = 0.17 wt.%, [photo‐initiator] = 0.25 wt.%, 4.5 mL) was poured into a glass container (diameter: 64 mm) and a photomask with a designed pattern (size: 50 × 50 × 0.1 mm; Figure S6, Supporting Information) was placed on the container. Then, the solution was irradiated with UV light through the photomask for 2.5 min by using an USHIO model OPM2‐502H super high‐pressure mercury lamp (500 W) to complete hydrogelation. After turning off the UV light and removing the photomask, the sample was left for 1.0 min. Then, the overall region of the sample was irradiated again with UV light for 2.5 min. Anisotropic hydrogels (Figure 5a‐i) were similarly synthesized. In Figure 5b, the periodicity of the photomask was varied (1, 2, and 3 mm). In Figure S8 (Supporting Information), anisotropic hydrogels with 3‐mm periodic patterns were synthesized by varying photo‐initiator concentration ([photo‐initiator] = 0, 0.025, 0.050, 0.10, and 0.25 wt.%) at a fixed UV irradiation time of 2.0 min for each exposure step and by varying the UV irradiation time (0, 1.0, 2.0, 3.0, and 4.0 min) at a fixed [photo‐initiator] of 0.050 wt.%. Intensity profiles (Figure 5b; Figure S8, Supporting Information) in the POM images were obtained by using ImageJ (Fiji).

Anisotropic hydrogels with chiral arrangements of polymer networks were typically synthesized by the following procedure (Figure 5c; Figure S9, Supporting Information). First, the bottom gel layer was prepared using a photomask with a rectangular pattern in the same manner. Then, the precursor solution ([DMAAm] = 17 wt.%, [BIS] = 0.17 wt.%, [photo‐initiator] = 0.25 wt.%) was poured onto the resultant hydrogel in the glass container, and the sample was irradiated with UV light through the photomask rotated 45° clockwise for 2.5 min. Subsequently, the photomask was removed, and the overall region of the sample was irradiated with UV light. By repeating these processes, an anisotropic hydrogel with a chiral arrangement of polymer networks (right‐handed gel in Figure 5c) was obtained. The opposite‐handed chiral arrangement was also realized by repeating the same processes in a counterclockwise manner (left‐handed gel in Figure 5c; Figure S9, Supporting Information).

## Conflict of Interest

The authors declare no conflict of interest.

## Author Contributions

T.T. and K.S. designed the experiments. T.T. performed all experiments. N.K. constructed the setup for measuring mechanical properties and facilitated the measurements. T.T. and K.S. analyzed the data and wrote the manuscript. All authors discussed the results and commented on the manuscript. K.S. conceived, designed, and supervised the project.

## Supporting information



Supporting Information

Supplementary Movie1

Supplementary Movie2

## Data Availability

The data that support the findings of this study are available from the corresponding author upon reasonable request.

## References

[1] Y. S. Zhang , A. Khademhosseini , Science 2017, 356, eaaf3627. 28473537 10.1126/science.aaf3627 PMC5841082

[2] K. Sano , Y. Ishida , T. Aida , Angew. Chem., Int. Ed. 2018, 57, 2532. 10.1002/anie.201708196 29034553

[3] E. Prince , E. Kumacheva , Nat. Rev. Mater. 2019, 4, 99.

[4] N. Khuu , S. Kheiri , E. Kumacheva , Trends Chem. 2021, 3, 1002.

[5] M. T. I. Mredha , I. Jeon , Prog. Mater. Sci. 2022, 124, 100870.

[6] Z. Chen , H. Wang , Y. Cao , Y. Chen , O. Akkus , H. Liu , C. Cao , Matter 2023, 6, 3803.

[7] M. T. I. Mredha , Y. Z. Guo , T. Nonoyama , T. Nakajima , T. Kurokawa , J. P. Gong , Adv. Mater. 2018, 30, 1704937. 10.1002/adma.201704937 29341264

[8] M. T. I. Mredha , H. H. Le , V. T. Tran , P. Trtik , J. Cui , I. Jeon , Mater. Horiz. 2019, 6, 1504

[9] Y. Ma , M. Hua , S. Wu , Y. Du , X. Pei , X. Zhu , F. Zhou , X. He , Sci. Adv. 2020, 6, eabd2520. 33208374 10.1126/sciadv.abd2520 PMC7673813

[10] M. Sun , H. Li , Y. Hou , N. Huang , X. Xia , H. Zhu , Q. Xu , Y. Lin , L. Xu , Sci. Adv. 2023, 9, eade6973. 36800416 10.1126/sciadv.ade6973 PMC9937573

[11] X. Sun , Y. Mao , Z. Yu , P. Yang , F. Jiang , Adv. Mater. 2024, 36, 2400084. 10.1002/adma.202400084 38517475

[12] Q. L. Zhu , C. Du , Y. Dai , M. Daab , M. Matejdes , J. Breu , W. Hong , Q. Zheng , Z. L. Wu , Nat. Commun. 2020, 11, 5166. 33056999 10.1038/s41467-020-18801-1 PMC7560679

[13] Q. L. Zhu , C. F. Dai , D. Wagner , M. Daab , W. Hong , J. Breu , Q. Zheng , Z. L. Wu , Adv. Mater. 2020, 32, 2005567. 10.1002/adma.202005567 33079426

[14] Q. L. Zhu , C. F. Dai , D. Wagner , O. Khoruzhenko , W. Hong , J. Breu , Q. Zheng , Z. L. Wu , Adv. Sci. 2021, 8, 2102353. 10.1002/advs.202102353 PMC8693068 34705341

[15] C. F. Dai , O. Khoruzhenko , C. Zhang , Q. L. Zhu , D. Jiao , M. Du , J. Breu , P. Zhao , Q. Zheng , Z. L. Wu , Angew. Chem., Int. Ed. 2022, 61, 202207272. 10.1002/anie.202207272 PMC9541020 35749137

[16] R. M. Erb , J. S. Sander , R. Grisch , A. R. Studart , Nat. Commun. 2013, 4, 1712. 23591879 10.1038/ncomms2666

[17] M. Liu , Y. Ishida , Y. Ebina , T. Sasaki , T. Hikima , M. Takata , T. Aida , Nature 2015, 517, 68. 25557713 10.1038/nature14060

[18] Y. S. Kim , M. Liu , Y. Ishida , Y. Ebina , M. Osada , T. Sasaki , T. Hikima , M. Takata , T. Aida , Nat. Mater. 2015, 14, 1002. 26259107 10.1038/nmat4363

[19] K. Sano , Y. O. Arazoe , Y. Ishida , Y. Ebina , M. Osada , T. Sasaki , T. Hikima , T. Aida , Angew. Chem., Int. Ed. 2018, 57, 12508. 10.1002/anie.201807240 30073724

[20] X. Wang , Z. Li , S. Wang , K. Sano , Z. Sun , Z. Shao , A. Takeishi , S. Matsubara , D. Okumura , N. Sakai , T. Sasaki , T. Aida , Y. Ishida , Science 2023, 380, 192. 37053325 10.1126/science.adf1206

[21] S. Bianco , F. Hallam Stewart , S. Panja , A. Zyar , E. Bowley , M. Bek , R. Kádár , A. Terry , R. Appio , T. S. Plivelic , M. Maguire , H. Poptani , M. Marcello , R. R. Sonani , E. H. Egelman , D. J. Adams , Nat. Synth. 2024, 3, 1481. 39664796 10.1038/s44160-024-00623-4 PMC11628395

[22] M. Chau , K. J. De France , B. Kopera , V. R. Machado , S. Rosenfeldt , L. Reyes , K. J. W. Chan , S. Förster , E. D. Cranston , T. Hoare , E. Kumacheva , Chem. Mater. 2016, 28, 3406.

[23] L. Li , Y. Zhang , H. Lu , Y. Wang , J. Xu , J. Zhu , C. Zhang , T. Liu , Nat. Commun. 2020, 11, 62. 31911636 10.1038/s41467-019-13959-9 PMC6946679

[24] M. Hua , S. Wu , Y. Ma , Y. Zhao , Z. Chen , I. Frenkel , J. Strzalka , H. Zhou , X. Zhu , X. He , Nature 2021, 590, 594. 33627812 10.1038/s41586-021-03212-z

[25] X. Guo , X. Dong , G. Zou , H. Gao , W. Zhai , Sci. Adv. 2023, 9, eadf7075. 36630512 10.1126/sciadv.adf7075 PMC9833652

[26] Z. Xu , H. Chen , H.‐B. Yang , X. Yao , H. Qin , H.‐P. Cong , S.‐H. Yu , Nat. Commun. 2025, 16, 400. 39755695 10.1038/s41467-024-55677-x PMC11700098

[27] M. Arifuzzaman , Z. L. Wu , R. Takahashi , T. Kurokawa , T. Nakajima , J. P. Gong , Macromolecules 2013, 46, 9083.

[28] R. Takahashi , Z. L. Wu , M. Arifuzzaman , T. Nonoyama , T. Nakajima , T. Kurokawa , J. P. Gong , Nat. Commun. 2014, 5, 4490. 25105259 10.1038/ncomms5490

[29] H. Guo , T. Nakajima , D. Hourdet , A. Marcellan , C. Creton , W. Hong , T. Kurokawa , J. P. Gong , Adv. Mater. 2019, 31, 1900702. 10.1002/adma.201900702 31074929

[30] Z. L. Wu , T. Kurokawa , S. Liang , H. Furukawa , J. P. Gong , J. Am. Chem. Soc. 2010, 132, 10064. 20590113 10.1021/ja101969k

[31] Z. L. Wu , T. Kurokawa , J. P. Gong , Polym. J. 2012, 44, 503.

[32] Y. Maki , K. Furusawa , T. Yamamoto , T. Dobashi , J. Biorheol. 2018, 32, 27.

[33] J. Nie , B. Pei , Z. Wang , Q. Hu , Carbohydr. Polym. 2019, 205, 225. 30446099 10.1016/j.carbpol.2018.10.033

[34] L. Qiao , C. Du , J. P. Gong , Z. L. Wu , Q. Zheng , Adv. Mater. Technol. 2019, 4, 1900665.

[35] W. Choi , M. Lee , H. Yong , D. Heo , T. Jun , H. Ryu , J.‐Y. Kim , D. Cui , D. Y. Ryu , S.‐Y. Lee , S.‐H. Choi , B.‐S. Kim , J. Kim , S. Y. Jung , S. Lee , J. Hong , Sci. Adv. 2024, 10, eadl3075. 38669324 10.1126/sciadv.adl3075 PMC11051667

[36] M. A. Haque , T. Kurokawa , J. P. Gong , Soft Matter 2012, 8, 8008.

[37] A. S. Gladman , E. A. Matsumoto , R. G. Nuzzo , L. Mahadevan , J. A. Lewis , Nat. Mater. 2016, 15, 413. 26808461 10.1038/nmat4544

[38] S. M. Chin , C. V. Synatschke , S. Liu , R. J. Nap , N. A. Sather , Q. Wang , Z. Álvarez , A. N. Edelbrock , T. Fyrner , L. C. Palmer , I. Szleifer , M. Olvera de la Cruz , S. I. Stupp , Nat. Commun. 2018, 9, 2395. 29921928 10.1038/s41467-018-04800-w PMC6008453

[39] N. Khuu , M. Alizadehgiashi , A. Gevorkian , E. Galati , N. Yan , E. Kumacheva , Adv. Mater. Technol. 2019, 4, 1800627.

[40] E. Prince , M. Alizadehgiashi , M. Campbell , N. Khuu , A. Albulescu , K. De France , D. Ratkov , Y. Li , T. Hoare , E. Kumacheva , Biomacromolecules 2018, 19, 1276. 29505709 10.1021/acs.biomac.8b00100

[41] J. Ma , S. Lin , Y. Jiang , P. Li , H. Zhang , Z. Xu , H. Wu , P. Lin , J. Breu , W. Gao , C. Gao , ACS Nano 2020, 14, 2336. 31951370 10.1021/acsnano.9b09503

[42] A. Gevorkian , S. M. Morozova , S. Kheiri , N. Khuu , H. Chen , E. Young , N. Yan , E. Kumacheva , Adv. Funct. Mater. 2021, 31, 2010743.

[43] S. Zhu , S. Wang , Y. Huang , Q. Tang , T. Fu , R. Su , C. Fan , S. Xia , P. S. Lee , Y. Lin , Nat. Commun. 2024, 15, 118. 38168050 10.1038/s41467-023-44481-8 PMC10761753

[44] Q. L. Zhu , W. Liu , O. Khoruzhenko , J. Breu , W. Hong , Q. Zheng , Z. L. Wu , Nat. Commun. 2024, 15, 300. 38182606 10.1038/s41467-023-44608-x PMC10770334

[45] J. Yang , R. Bai , B. Chen , Z. Suo , Adv. Funct. Mater. 2020, 30, 1901693.

[46] G. Bovone , O. Y. Dudaryeva , B. Marco‐Dufort , M. W. Tibbitt , ACS Biomater. Sci. Eng. 2021, 7, 4048. 33792286 10.1021/acsbiomaterials.0c01677

[47] Z. Hu , X. Zhang , Y. Li , Science 1995, 269, 525. 17842364 10.1126/science.269.5223.525

[48] A. Harada , R. Kobayashi , Y. Takashima , A. Hashidzume , H. Yamaguchi , Nat. Chem. 2011, 3, 34. 21160514 10.1038/nchem.893

[49] B. C. Zarket , S. R. Raghavan , Nat. Commun. 2017, 8, 193. 28779112 10.1038/s41467-017-00077-7 PMC5544678

[50] S. Xiao , Y. Yang , M. Zhong , H. Chen , Y. Zhang , J. Yang , J. Zheng , ACS Appl. Mater. Interfaces 2017, 9, 20843. 28570039 10.1021/acsami.7b04417

[51] D. Wirthl , R. Pichler , M. Drack , G. Kettlguber , R. Moser , R. Gerstmayr , F. Hartmann , E. Bradt , R. Kaltseis , C. M. Siket , S. E. Schausberger , S. Hild , S. Bauer , M. Kaltenbrunner , Sci. Adv. 2017, 3, 1700053. 10.1126/sciadv.1700053 PMC5479648 28691092

[52] J. Yang , R. Bai , Z. Suo , Adv. Mater. 2018, 30, 1800671. 10.1002/adma.201800671 29726051

[53] J. Steck , J. Yang , Z. Suo , ACS Macro Lett. 2019, 8, 754. 35619535 10.1021/acsmacrolett.9b00325

[54] S. Tamesue , T. Endo , Y. Ueno , F. Tsurumaki , Macromolecules 2019, 52, 5690.

[55] F. G. Downs , D. J. Lunn , M. J. Booth , J. B. Sauer , W. J. Ramsay , R. G. Klemperer , C. J. Hawker , H. Bayley , Nat. Chem. 2020, 12, 363. 32221498 10.1038/s41557-020-0444-1 PMC7117959

[56] J. Li , Q. Ma , Y. Xu , M. Yang , Q. Wu , F. Wang , P. Sun , ACS Appl. Mater. Interfaces 2020, 12, 55290. 33232107 10.1021/acsami.0c17085

[57] X. He , S. Wang , J. Zhou , D. Zhang , Y. Xue , X. Yang , L. Che , D. Li , S. Xiao , S. Liu , S. Y. Zheng , J. Yang , ACS Appl. Mater. Interfaces 2022, 14, 4579. 35029363 10.1021/acsami.1c22887

[58] Q. Mu , K. Cui , Z. J. Wang , T. Matsuda , W. Cui , H. Kato , S. Namiki , T. Yamazaki , M. Frauenlob , T. Nonoyama , M. Tsuda , S. Tanaka , T. Nakajima , J. P. Gong , Nat. Commun. 2022, 13, 6213. 36266283 10.1038/s41467-022-34044-8 PMC9585076

[59] S. Ye , W. Ma , G. Fu , Chem. Mater. 2023, 35, 999.

[60] F. Chen , X. Li , Y. Yu , Q. Li , H. Lin , L. Xu , H. C. Shum , Nat. Commun. 2023, 14, 2793. 37193701 10.1038/s41467-023-38394-9 PMC10188440

[61] D. Zhang , Y. Tang , K. Zhang , Y. Xue , S. Y. Zheng , B. Wu , J. Zheng , Matter 2023, 6, 1484.

[62] S. Ishikawa , Y. Iwanaga , T. Uneyama , X. Li , H. Hojo , I. Fujinaga , T. Katashima , T. Saito , Y. Okada , U.‐i. Chung , N. Sakumichi , T. Sakai , Nat. Mater. 2023, 22, 1564. 37903925 10.1038/s41563-023-01712-z

[63] J. Liu , Y.‐S. Huang , Y. Liu , D. Zhang , K. Koynov , H.‐J. Butt , S. Wu , Nat. Chem. 2024, 16, 1024. 38459235 10.1038/s41557-024-01476-2 PMC11164683

[64] D. L. Taylor , M. in het Panhuis , Adv. Mater. 2016, 28, 9060. 27488822 10.1002/adma.201601613

[65] J. Liu , M. W. Urban , Langmuir 2024, 40, 7268. 38395626 10.1021/acs.langmuir.3c03696

[66] H. Qin , T. Zhang , N. Li , H.‐P. Cong , S.‐H. Yu , Nat. Commun. 2019, 10, 2202. 31101823 10.1038/s41467-019-10243-8 PMC6525195

[67] J. Jia , S. Lu , S. Sun , Y. Jin , L. Qin , C. Zhao , Sci. Adv. 2025, 11, eadr9834. 39854461 10.1126/sciadv.adr9834 PMC11759658

[68] Q. Ge , Z. Chen , J. Cheng , B. Zhang , Y.‐F. Zhang , H. Li , X. He , C. Yuan , J. Liu , S. Magdassi , S. Qu , Sci. Adv. 2021, 7, eaba4261. 33523958 10.1126/sciadv.aba4261 PMC7787492

[69] B. Liu , H. Li , F. Meng , Z. Xu , L. Hao , Y. Yao , H. Zhu , C. Wang , J. Wu , S. Bian , W. W. Lu , W. Liu , H. Pan , X. Zhao , Nat. Commun. 2024, 15, 1587. 38383668 10.1038/s41467-024-45938-0 PMC10881973

[70] M. Caprioli , I. Roppolo , A. Chiappone , L. Larush , C. F. Pirri , S. Magdassi , Nat. Commun. 2021, 12, 2462. 33911075 10.1038/s41467-021-22802-z PMC8080574

[71] Z. U. Arif , M. Y. Khalid , A. Tariq , M. Hossain , R. Umer , Giant 2024, 17, 100209.

[72] C. Y. Li , X. P. Hao , Z. L. Wu , Q. Zheng , Chem. Asian J. 2019, 14, 94. 30239161 10.1002/asia.201801333

[73] J. Kim , J. A. Hanna , M. Byun , C. D. Santangelo , R. C. Hayward , Science 2012, 335, 1201. 22403385 10.1126/science.1215309

[74] H. Thérien‐Aubin , Z. L. Wu , Z. Nie , E. Kumacheva , J. Am. Chem. Soc. 2013, 135, 4834. 23464872 10.1021/ja400518c

[75] Z. L. Wu , M. Moshe , J. Greener , H. Therien‐Aubin , Z. Nie , E. Sharon , E. Kumacheva , Nat. Commun. 2013, 4, 1586. 23481394 10.1038/ncomms2549

[76] Z. J. Wang , C. N. Zhu , W. Hong , Z. L. Wu , Q. Zheng , Sci. Adv. 2017, 3, 1700348. 10.1126/sciadv.1700348 PMC5600534 28929134

[77] F. Huang , M. Chen , Z. Zhou , R. Duan , F. Xia , I. Willner , Nat. Commun. 2021, 12, 2364. 33888708 10.1038/s41467-021-22645-8 PMC8062675

[78] P. Fandrich , L. Wiehemeier , M. Dirksen , O. Wrede , T. Kottke , T. Hellweg , Colloid Polym. Sci. 2021, 299, 221.

[79] L. Tang , L. Wang , X. Yang , Y. Feng , Y. Li , W. Feng , Prog. Mater. Sci. 2021, 115, 100702.

[80] H. Feng , N. Tang , M. An , R. Guo , D. Ma , X. Yu , J. Zang , N. Yang , J. Phys. Chem. C 2019, 123, 31003.

[81] M. An , B. Demir , X. Wan , H. Meng , N. Yang , T. R. Walsh , Adv. Theory Simul. 2019, 2, 1800153.

