# Supplementary material for: Gel‐Gel Interface Engineering for the Synthesis of Anisotropic Hydrogels with Designable Polymer Orientations
Source: Adv Mater. 2025 Jul 9;37(43):2505268. doi: 10.1002/adma.202505268 (PMC12574624; doi:10.1002/adma.202505268)
Supplement: Supplementary file 1 — Supporting Information [file ADMA-37-2505268-s003.pdf]

ADVANCED MATERIALS

Supporting Information

for *Adv. Mater.*, DOI 10.1002/adma.202505268

Gel-Gel Interface Engineering for the Synthesis of Anisotropic Hydrogels with Designable Polymer Orientations

*Tomohiro Takahashi, Naoya Karasawa and Koki Sano**

Supporting Information

Gel-Gel Interface Engineering for the Synthesis of Anisotropic Hydrogels with Designable Polymer Orientations

*Tomohiro Takahashi, Naoya Karasawa, and Koki Sano**

Department of Chemistry and Materials, Faculty of Textile Science and Technology, Shinshu University, 3-15-1 Tokida, Ueda, Nagano 386-8567, Japan.

E-mail: koki_sano@shinshu-u.ac.jp

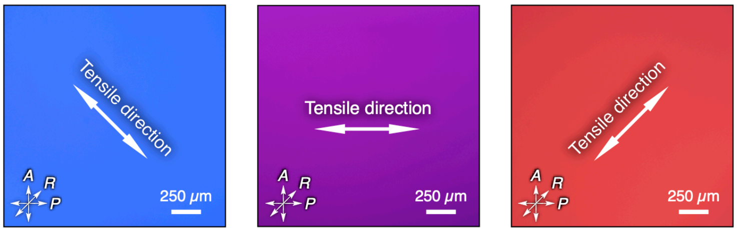

Figure S1. Polarized optical microscopy images under crossed Nicols with a sensitive tint plate of an elongated hydrogel (composition: [DMAAm] = 10 wt%, [BIS] = 0.10 wt%, [APS] = 2.3 wt%, [TEMED] = 0.2 wt%; tensile strain: ~70%) at different rotation angles.

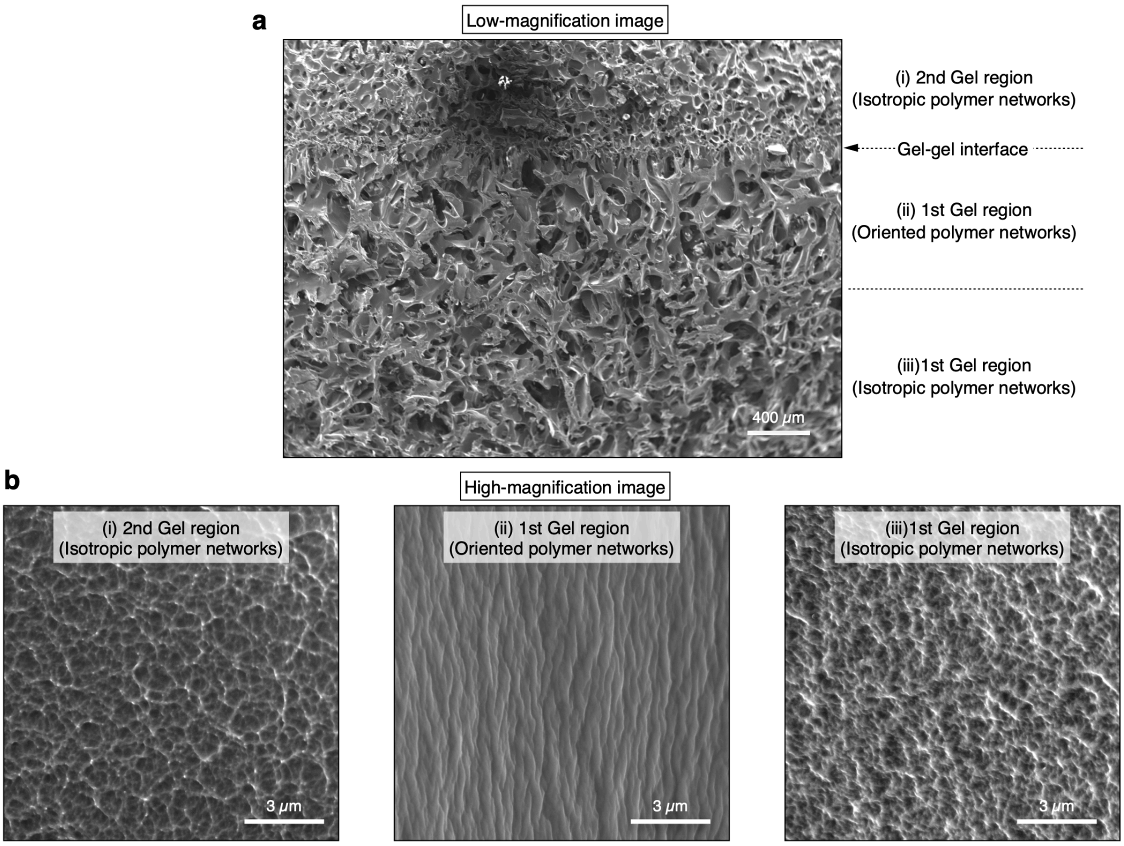

Figure S2. a,b) Field-emission scanning electron microscopy (FE-SEM) images of a dried anisotropic hydrogel around the gel-gel interface at low (a) and high (b) magnifications. The high-magnification images (b) were taken from the three distinct regions indicated in (a): (i) the 2nd gel region with isotropic polymer networks, (ii) the 1st gel region with oriented polymer networks, and (iii) the 1st gel region with isotropic polymer networks.

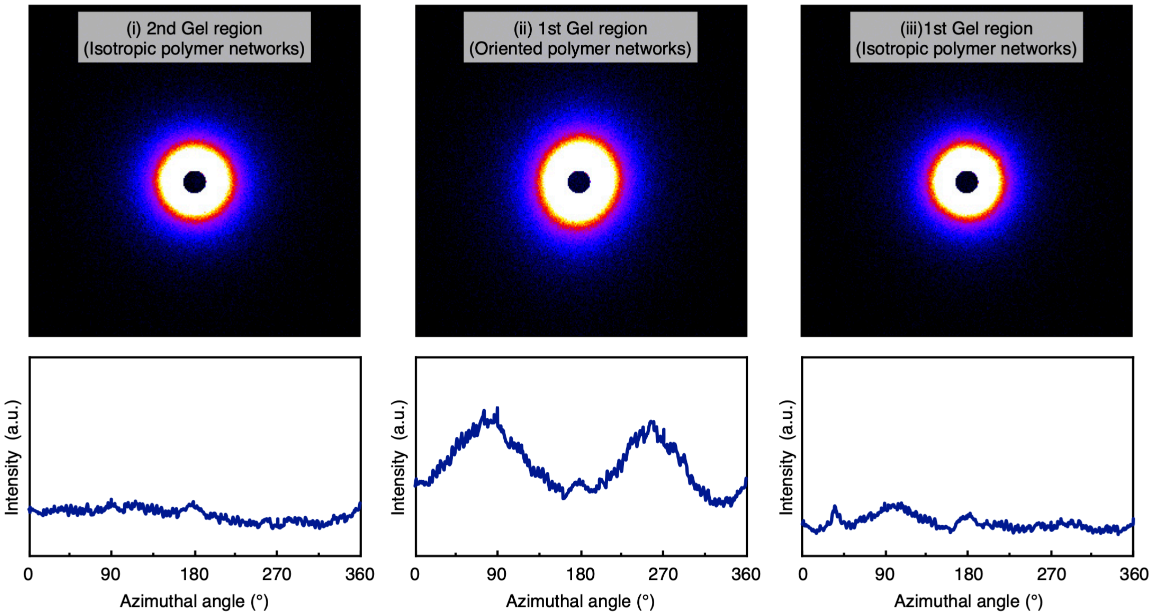

Figure S3. Two-dimensional small-angle X-ray scattering (2D-SAXS) patterns (upper) and the corresponding azimuthal angle plots (lower) of a dried anisotropic hydrogel in the three distinct regions around the gel-gel interface: (i) the 2nd gel region with isotropic polymer networks, (ii) the 1st gel region with oriented polymer networks, and (iii) the 1st gel region with isotropic polymer networks.

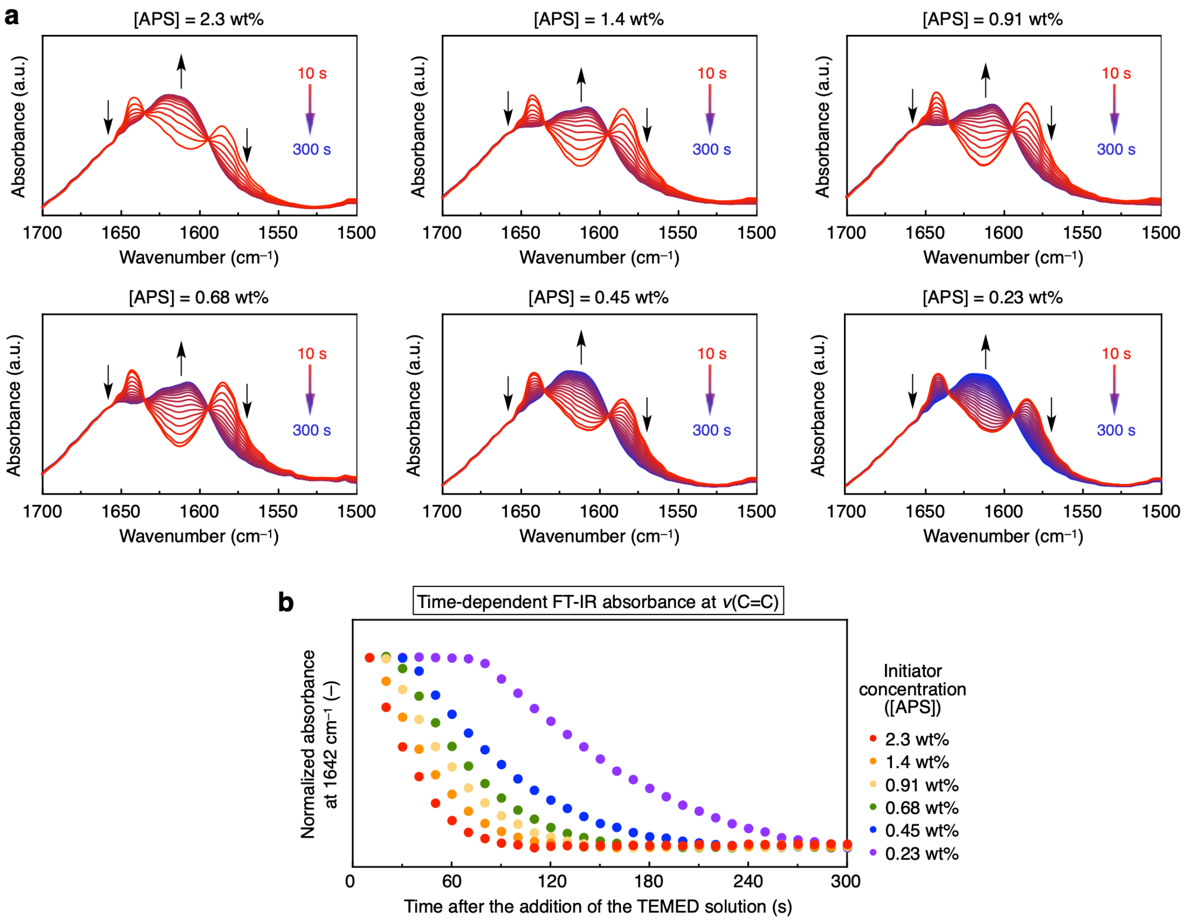

Figure S4. a,b) Time-dependent changes in attenuated total reflectance (ATR) FT-IR spectra (a) and the normalized absorbance at 1642 cm^{-1} (b), corresponding to the C=C stretching vibration (v(C=C)) of the monomer (DMAAm) during gelation of the precursor solutions with different initiator concentrations ([APS]) (composition: [DMAAm] = 10 wt%, [BIS] = 0.10 wt%, [APS] = 0.23, 0.45, 0.68, 0.91, 1.4, and 2.3 wt%, [TEMED] = 0.2 wt%) after the addition of TEMED solution to accelerate radical polymerization. The ATR FT-IR measurements during gelation were performed using a JASCO model FT/IR-6600 spectrometer.

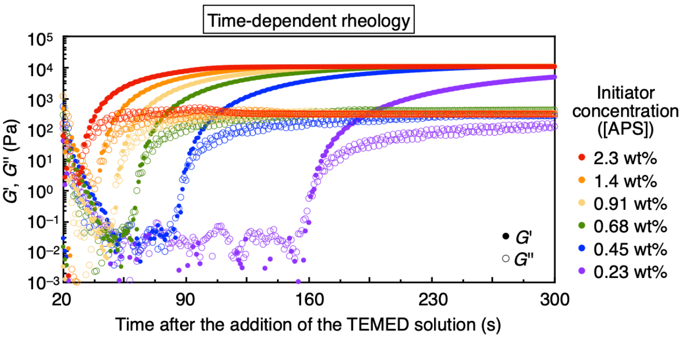

Figure S5. Time-dependent changes in the storage (G') and loss (G'') moduli during gelation of the precursor solutions with different initiator concentrations ([APS]) (composition: [DMAAm] = 10 wt%, [BIS] = 0.10 wt%, [APS] = 0.23, 0.45, 0.68, 0.91, 1.4, and 2.3 wt%, [TEMED] = 0.2 wt%) after the addition of TEMED solution to accelerate radical polymerization. The rheological measurements during gelation were carried out at 25 °C using an Anton Paar model MCR-302e rheometer at fixed conditions (frequency: 1 rad s^{-1}; strain: 1%; parallel plate diameter: 25 mm; sample gap: 2 mm).

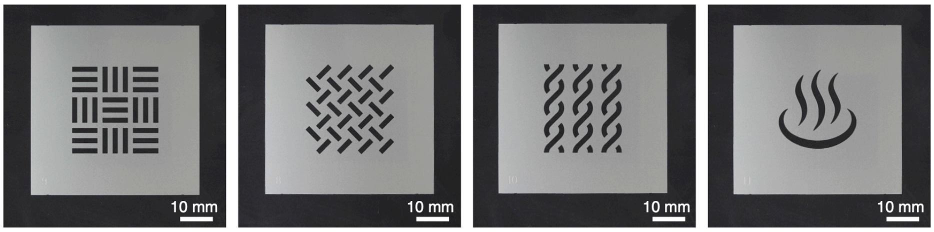

Figure S6. Photographs of photomasks with designed patterns used for the synthesis of anisotropic hydrogels with designable polymer orientations.

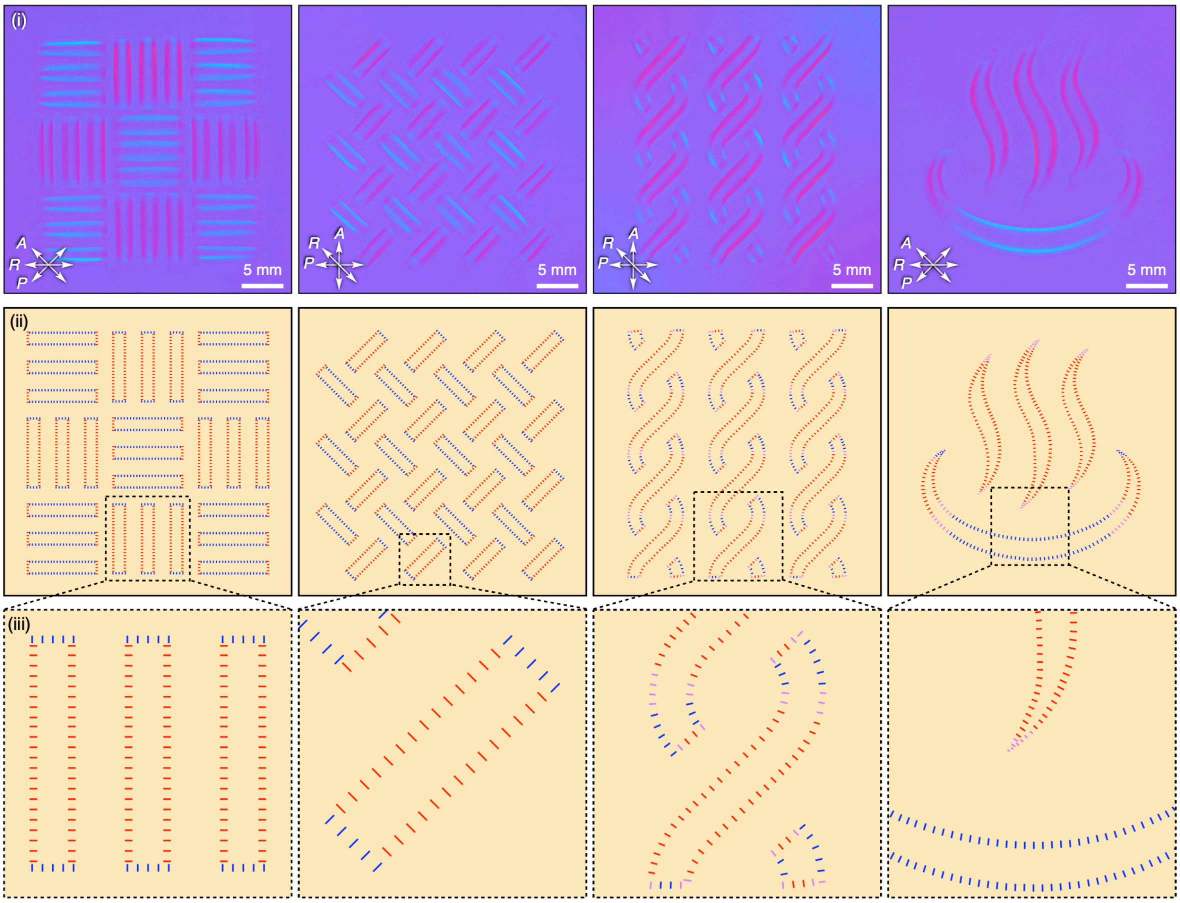

Figure S7. Polymer orientations in anisotropic hydrogels. (i) Polarized optical top-view images under crossed Nicols with a sensitive tint plate of the anisotropic hydrogels with designed polymer orientations (same images as Figure 5a-iv). (ii) Schematic illustrations of polymer orientations in the anisotropic hydrogels. The local orientations of the polymer networks align parallel to the short lines in the images. (iii) Magnified views of (ii).

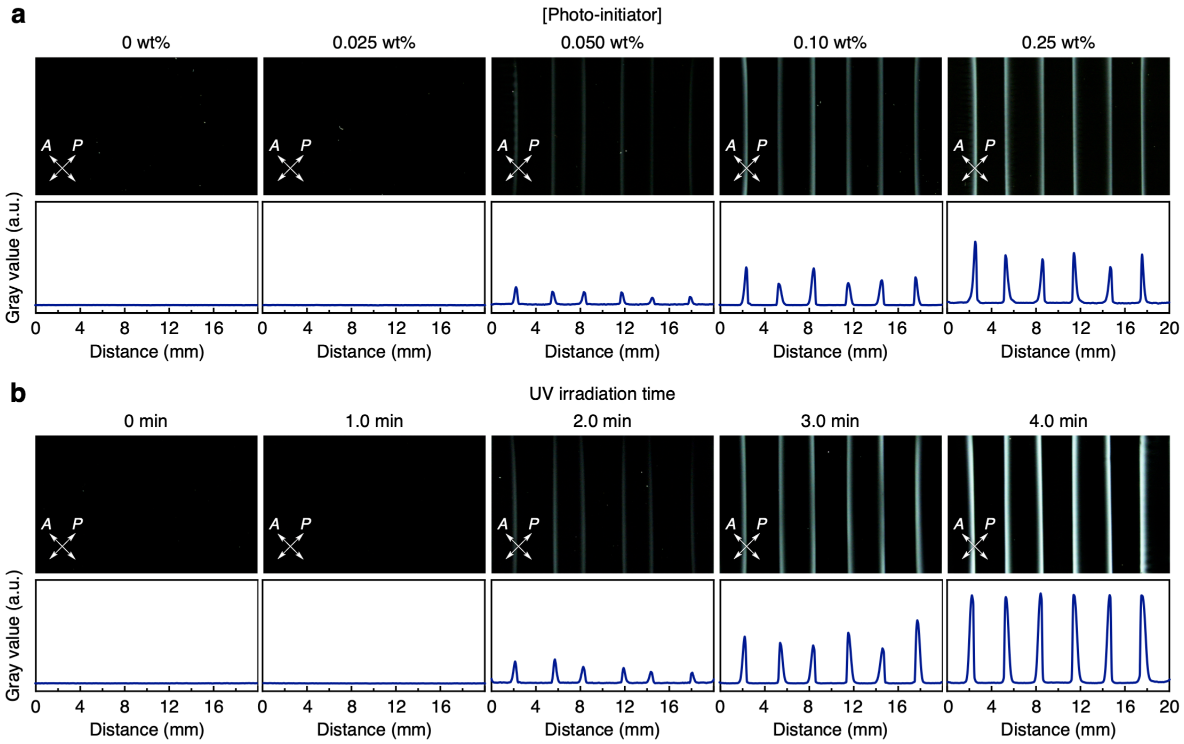

Figure S8. Effects of UV irradiation conditions on periodic patterning of hydrogels. a,b) Polarized optical images (upper) and the corresponding gray value profiles (lower) of anisotropic hydrogels with 3-mm periodic patterns synthesized by varying photo-initiator concentration ([photo-initiator] = 0, 0.025, 0.050, 0.10, and 0.25 wt%) at a fixed UV irradiation time of 2.0 min for each step (a) and by varying the UV irradiation time (0, 1.0, 2.0, 3.0, and 4.0 min) at a fixed [photo-initiator] of 0.050 wt% (b).

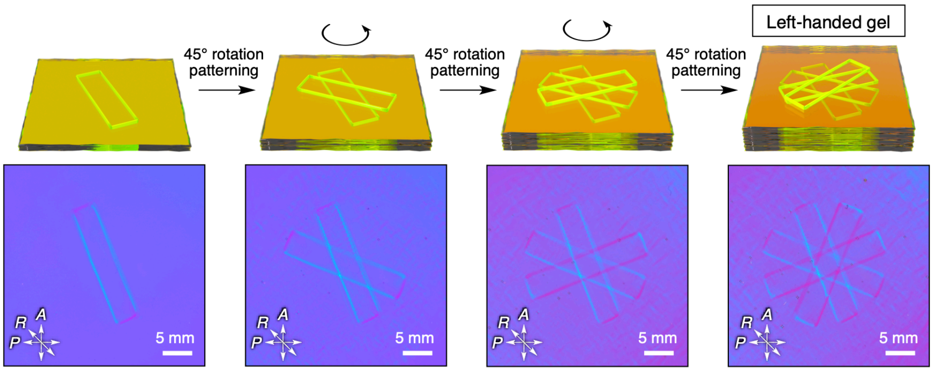

Figure S9. Polarized optical images under crossed Nicols with a sensitive tint plate of anisotropic hydrogels with chiral arrangements of polymer networks (a left-handed gel) synthesized via a step-by-step photo-patterning process.

Movie S1. A POM movie (600× speed) under crossed Nicols of the gel-gel interface after the addition of a precursor solution for the 2nd gel without the radical initiator (APS/TEMED).

Movie S2. A POM movie (200× speed) under crossed Nicols of the gel-gel interface after the addition of a precursor solution for the 2nd gel with the radical initiator (APS/TEMED).
